# Predicting the European stock market during COVID-19: A machine learning approach

**DOI:** 10.1016/j.mex.2020.101198

**Published:** 2020-12-23

**Authors:** Mudeer Ahmed Khattak, Mohsin Ali, Syed Aun R. Rizvi

**Affiliations:** a Lahore University of Management Sciences, Pakistan; b Iqra University, Islamabad Campus, Pakistan; c Taylor's University, Malaysia

**Keywords:** Europe, Stock markets, Coronavirus, Least Absolute Shrinkage and Selection Operator (LASSO)

## Abstract

This research attempts to explore the total of 21 potential internal and external shocks to the European market during the Covid-19 Crisis. Using the time series of 1 Jan 2020 to 26 June 2020, I employ a machine learning technique, i.e. Least Absolute Shrinkage and Selection Operator (LASSO) to examine the research question for its benefits over the traditional regression methods. This further allows me to cater to the issue of limited data during the crisis and at the same time, allows both variable selection and regularization in the analysis. Additionally, LASSO is not susceptible to and sensitive to outliers and multi-collinearity. The European market is mostly affected by indices belonging to Singapore, Switzerland, Spain, France, Germany, and the S&P500 index. There is a significant difference in the predictors before and after the pandemic announcement by WHO. Before the Pandemic period announcement by WHO, Europe was hit by the gold market, EUR/USD exchange rate, Dow Jones index, Switzerland, Spain, France, Italy, Germany, and Turkey and after the announcement by WHO, only France and Germany were selected by the lasso approach. It is found that Germany and France are the most predictors in the European market.

• A LASSO approach is used to predict the European stock market index during COVID-19
• European market is mostly affected by the indices belonging to Singapore, Switzerland, Spain, France, Germany, and the S&P500 index.
• There is a significant difference in the predictors before and after the pandemic announcement by WHO.

## Graphical abstract

**Figure:**
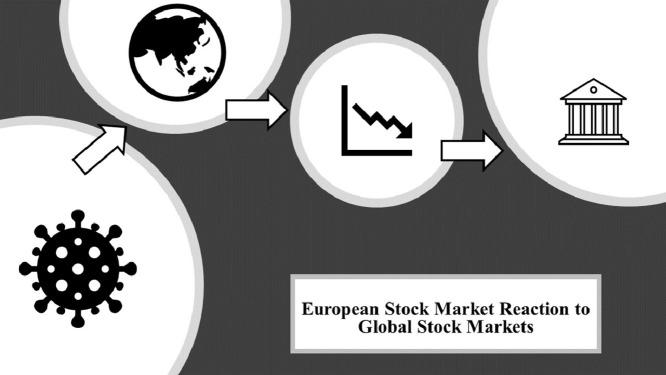

Specifications table

**Table:** 

Subject Area	*Economics and Finance*
More specific subject area	*Stock Markets*
Method name	*Least Absolute Shrinkage and Selection Operator (LASSO) Regression*
Name and reference of original method	*Tibshirani, Robert. 1996. “Regression Shrinkage and Selection Via the Lasso.” Journal of the Royal Statistical Society: Series B (Methodological).* *Tibshirani, Robert. 2011. “Regression Shrinkage and Selection via the Lasso: A Retrospective.” Journal of the Royal Statistical Society. Series B: Statistical Methodology.* *Hastie, Trevor, Robert Tibshirani, and Martin Wainwright (2015). Statistical Learning with Sparsity: The Lasso and Generalizations.*
Resource availability	*DATASTREAM* *STATA 16.0*

## Introduction

The world has seen enough of the catastrophic implication of the COVID-19. Till 27 June, confirmed COVID-19 infections have crossed 10 million and with the loss of 5,02,208 lives around the globe. This had a further devastating impact on the global economy (See: [1], [2], [3]). Global Financial markets are also not spared, and the impact is destructive to the overall global financial markets.

**Fig. 1:**
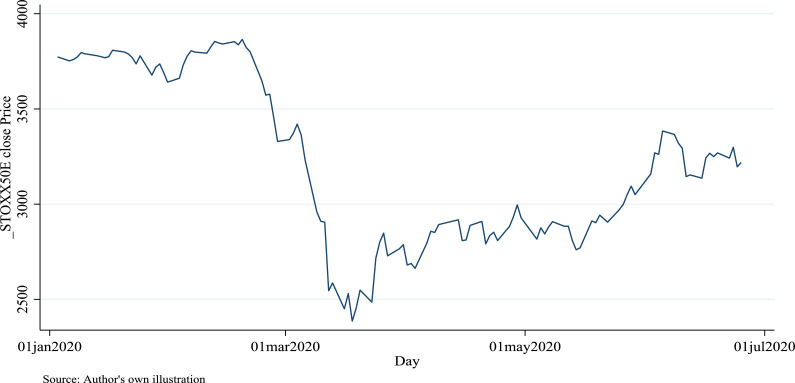
EURO STOX 50 index.

The international market integration has been a topic of interest in recent times. The coronavirus has had a significant impact on the global economy. This increased volatility and impact on the world financial market is well documented in the literature [4], [5], [6] and in literature focusing on regional financial markets as well [7], [8], [9], [10], [11], [12]. Fig. 1 shows the movement of the Euro STOXX-50 index throughout the sampling period. Since all the markets around the globe have been experiencing shocks, it is not clear which market, inside or outside Europe, played a role in affecting the European market.

The literature on pandemic and financial markets is evolving and currently explores rising volatility due to COVID-19 and uncertainty in the financial markets (see [13], [14], [15], [16]).

In this study, we analyze the potential European and global markets that shook Europe. To do this, 21 different potential predictors of the European financial market including predictors inside and outside Europe are used. The 21 indicators include indices from countries which got impacted by the COVID-19 the most along with gold, oil and bitcoins. This is done using a machine learning approach named LASSO regression, which allows both the model selection and regularization in the analysis. Germany, France, Gold, the S&P500, Singapore, Switzerland, Spain, Italy, Turkey, the Dow Jones, and the EUR/USD are found to be among the most important predictors of Europe. Germany and France are found to be the top predictors of Europe.

The remainder of the article is structured as follows: the following section provides information on data and methods which describe the data sources, the variables specification, and estimation methods. This followed by a detailed discussion on the analysis and discussion on the importance of the predictors found to be important to Europe. Finally, the conclusion section.

## Data and methods

We employ daily data from 1 January 2020 till 26 June 2020^1^ sourced from various sources having (153 observations). We consider the full period as well as two sub-periods separately: First, sub-period is before the WHO announcement of a global pandemic, from 1 Jan to 10th march, and second, after the WHO declared a pandemic on 11th March. This second period is from 11th March to 26 June. We use the EURO STOXX 50 index as the dependent variable for all the models.

We employ a machine learning technique, known as the Least Absolute Shrinkage and Selection Operator (LASSO) for the default sample and subsample periods. The literature on LASSO provides two main advantages of employing lasso over other methodologies like wavelet or ANN; first, LASSO offers a special feature of coefficient shrinkage, which automates the model selection in linear regression due to the nature of 1-penalty, and while doing so it further removes some variables from the model. It also factors in the the issue of multicollinearity as it is not sensitive to outliers and multicollinearity [17].

LASSO combines the least-squares estimator with an extra constraint on the sum of the absolute values of the coefficients. The limited data availability, during the COVID-19 crisis, might be an issue for traditional times series analysis, however, learning from the data, lasso also takes care of this problem and gives the best predictors.

(1) $$\beta^{\text{Lasso}\left(\lambda \right)}=\text{Argmin}\frac{1}{n}\sum_{j=1}^{n}{\left(y_{j\;}-{x^{\prime }}_{j}\beta \right)}^{2}+\frac{\lambda }{n}\sum_{i=1}^{p}\gamma_{i}\left|\beta_{i}\right|$$

Where λ indicates the tuning parameter, which regulates the overall penalty level and $\gamma_{i}\;$are predictor-specific penalty loadings. The lasso coefficient path, which signifies the trajectory of the estimated coefficient as a function of λ, is piecewise linear with changes in slope where the variable enters or leaves the active set. If λ = 0, it gives the OLS solution and λ approaching to ∞ gives an empty model, where all coefficients are zero. For more on LASSO see [18], [19], [20], [21].

## Results and discussion

Table 1 presents the estimated coefficients of the selected predictors for the default sample, before the pandemic, after the pandemic announcement by WHO. It appears that not all the predictors have similar control over Europe across different periods and the predictor selection changes across different subsamples. For the full sample, the most important indices affecting the European financial market are the S&P500 and the indices of Singapore, Switzerland, Spain, France, and Germany.

**Table 1:** Coefficients of the independent variables for each model.

Full Period (1 Jan-31 May)		Before Pandemic (1 Jan-10 March)		Pandemic (11 March-25 June)	
Variable	lasso Coefficient	Variable	lasso Coefficient	Variable	lasso Coefficient
S&P500	−0.011	GOLD	−0.067	France	0.599
Singapore	−0.038	EUSDD	0.394	Germany	0.392
Switzerland	0.058	DJ	0.012		
Spain	0.067	Switzerland	0.072		
France	0.517	Spain	0.203		
Germany	0.403	France	0.495		
		Italy	0.102		
		Germany	0.080		
		Turkey	0.067		
_cons	0.010	_cons	0.108	_cons	0.049

Note: The LASSO model is estimated for the full sample and two subsamples. LASSO only selects and reports the variables that are important to the dependent variable and LASSO drops the variables that are not important from the model.

Before the pandemic announcement, Gold, EUSDD, DJ, Switzerland, Spain, France, Italy, Germany, and Turkey indices are found to be the most important commodities and indices that impacted Europe. For the third sample, that is after the announcement of the global pandemic by WHO, only France and Germany are found to be impacting Europe. However, no commodity market is found to be impacting Europe. The plausible reason could be that oil prices were falling due to its own dynamics [22]. Secondly, oil and gold showed inefficient behavior during the period understudy [23]

**Fig. 2:**
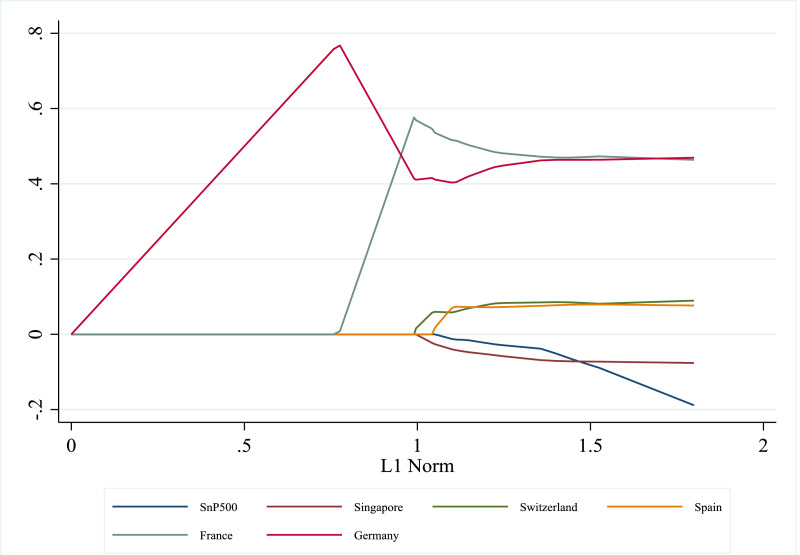
LASSO Coefficient paths for default Period (1 Jan-26 June).

**Fig. 3:**
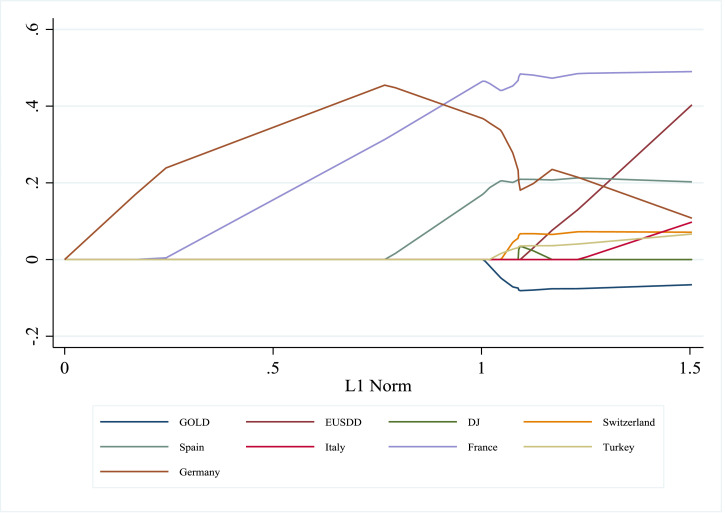
LASSO Coefficient paths for Before Pandemic (1 Jan-10 March).

**Fig. 4:**
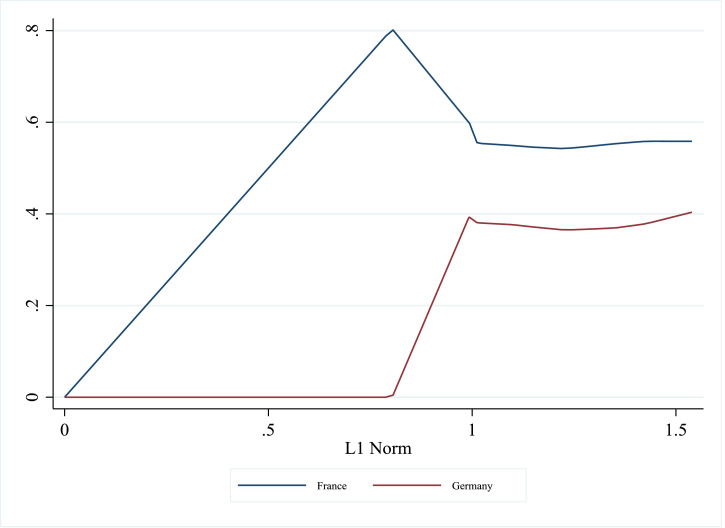
LASSO Coefficient paths for After Pandemic (11 March-26 June).

To get further insights into the degree of importance of the selected predictors, the coefficient paths obtained from models are plotted in Figs. 2–4. The magnitude of the coefficient is shown on the *y*-axis and the L1 norm is shown on the *x*-axis. For the default sample, it is found that all the selected predictors shown in Table 1 show a significant impact on the European market. the first covariate that shows any impact and diverges first from zero is Germany, followed by France, Switzerland, Singapore, Spain, and S&P500. This indicates that Germany and France are of the most important predictors of the European Market. For the period before the pandemic, Germany, and France, once again, show the most importance. This is followed by Spain and Gold. Turkey, Switzerland, Dow Jones. EUR/USD and Italy are among the last covariates to diverge. For the period of the Pandemic, only Germany and France are found to be the predictors of the European market. This further strengthens the earlier finding that Germany and France are potential predictors of the European market and these two markets are shaping the overall European market. The plausible reasons could be, first, they are two of the strongest stock markets in Europe [24]. Secondly, these two were also the most affected countries from COVID-19.

Addressing the nature of the impact of these predictors, these figures suggest that overall, the impact on Europe is (1) negative from Gold, the S&P500, and Singapore. And (2) positive from, along with the indices from, Switzerland, Spain, Italy, Turkey, the Dow Jones, and the EUR/USD. While Germany and France are found to be the top positive predictors of Europe. Though the LASSO selects different variables across different subsamples, the impact of selected variables remains unchanged.

## Conclusion

We attempted to explore the potential predictors of the European financial market by examining the impact of protentional internal and external determinants. Germany, France, Gold prices, S&P500, Singapore, Switzerland, Spain, Italy, Turkey, Dow Jones, and the EUR/USD are found to be the predictors of the Europe market. Germany and France are found to be the most important determinants of the European market. Though the LASSO selects different variables across different subsamples, the impact of selected variables remains unchanged.

## Declaration of Competing Interest

The authors declare that they have no known competing financial interests or personal relationships that could have appeared to influence the work reported in this paper.

## Appendix Supplementary materials

Image, application 1

## Footnotes

1 Period until June, 2020 was the most uncertain period as far as COVID-19 is concerned (Ngonghala et al. 2020).

## References

[1] Haroon O., Rizvi S.A.R. (2020). Flatten the curve and stock market liquidity—an inquiry into emerging economies. Emerg. Markets Financ. Trade.

[2] Haroon O., Rizvi S.A.R. (2020). COVID-19: Media coverage and financial markets behavior—a sectoral inquiry. J. Behav. Exp. Financ..

[3] Narayan P.K. (2020). Oil price news and COVID-19—is there any connection?. Energy Res. Lett..

[4] He P., Niu H., Sun Z., Li T. (2020). Accounting index of COVID-19 impact on Chinese industries: a case study using big data portrait analysis. Emerg. Markets Financ. Trade.

[5] Chen C., Liu L., Zhao N. (2020). Fear sentiment, uncertainty, and bitcoin price dynamics: the case of COVID-19. Emerg. Markets Financ. Trade.

[6] Phan D.H.B., Narayan P.K. (2020). Country responses and the reaction of the stock market to COVID-19—a preliminary exposition. Emerg. Markets Financ. Trade.

[7] Narayan P.K. (2020). Did bubble activity intensify during COVID-19?. Asian Econ. Lett..

[8] Narayan P.K. (2020). Has COVID-19 changed exchange rate resistance to shocks. Asian Econ. Lett..

[9] Sharma S.S. (2020). A note on the Asian market volatility during the COVID-19 pandemic. Asian Econ. Lett..

[10] Iyke B.N. (2020). COVID-19: the reaction of US oil and gas producers to the pandemic. Energy Res. Lett..

[11] Iyke B.N. (2020). The disease outbreak channel of exchange rate return predictability: evidence from COVID-19. Emerg. Markets Financ. Trade.

[12] Iyke B.N. (2020). Economic policy uncertainty in times of COVID-19 pandemic. Asian Econ. Lett..

[13] Ali M., Alam N., Rizvi S.A.R. (2020). Coronavirus (COVID-19) – an epidemic or pandemic for financial markets. J. Behav. Exp. Financ..

[14] Apergis E., Apergis N. (2020). Can the COVID-19 pandemic and oil prices drive the US Partisan Conflict Index?. Energy Res. Lett..

[15] Devpura N., Narayan P.K. (2020). Hourly oil price volatility: The role of COVID-19. Energy Res. Lett..

[16] Gil-Alana L.A., Monge M. (2020). Crude oil prices and COVID-19: persistence of the shock. Energy Res. Lett..

[17] Colak G., Fu M., Hasan I. (2020). Why are some Chinese firms failing in the US capital markets? A Machine Learning Approach. Pac.-Basin Financ. J..

[18] Huang W., Zheng Y. (2020). COVID-19: structural changes in the relationship between investor sentiment and crude oil futures price. Energy Res. Lett..

[19] Liu L., Wang E.Z., Lee C.C. (2020). Impact of the COVID-19 pandemic on the crude oil and stock markets in the US: a time-varying analysis. Energy Res. Lett..

[20] Hastie T., Tibshirani R., Friedman J. (2009). Springer Series in Statistics the Elements of Statistical Learning – Data Mining, Inference, and Prediction.

[21] Hastie T., Tibshirani R., Wainwright M. (2015). Statistical Learning with Sparsity: the Lasso and Generalizations. Statistical Learning with Sparsity: The Lasso and Generalizations.

[22] Tibshirani R. (1996). Regression shrinkage and selection via the Lasso. J. R. Stat. Soc..

[23] Mensi W., Sensoy A., Vo X.V., Kang S.H. (2020). Impact of COVID-19 outbreak on asymmetric multifractality of gold and oil prices. Resour. Policy.

[24] Prabheesh K.P., Padhan R., Garg B. (2020). COVID-19 and the oil price–stock market nexus: evidence from net oil-importing countries. Energy Res. Lett..

