# Supplementary figures and images for: Predicting the European stock market during COVID-19: A machine learning approach
Source: MethodsX. 2020 Dec 23;8:101198. doi: 10.1016/j.mex.2020.101198 (PMC7777545; doi:10.1016/j.mex.2020.101198)

Figure A1: Movement of the Global financial markets during Covid-19

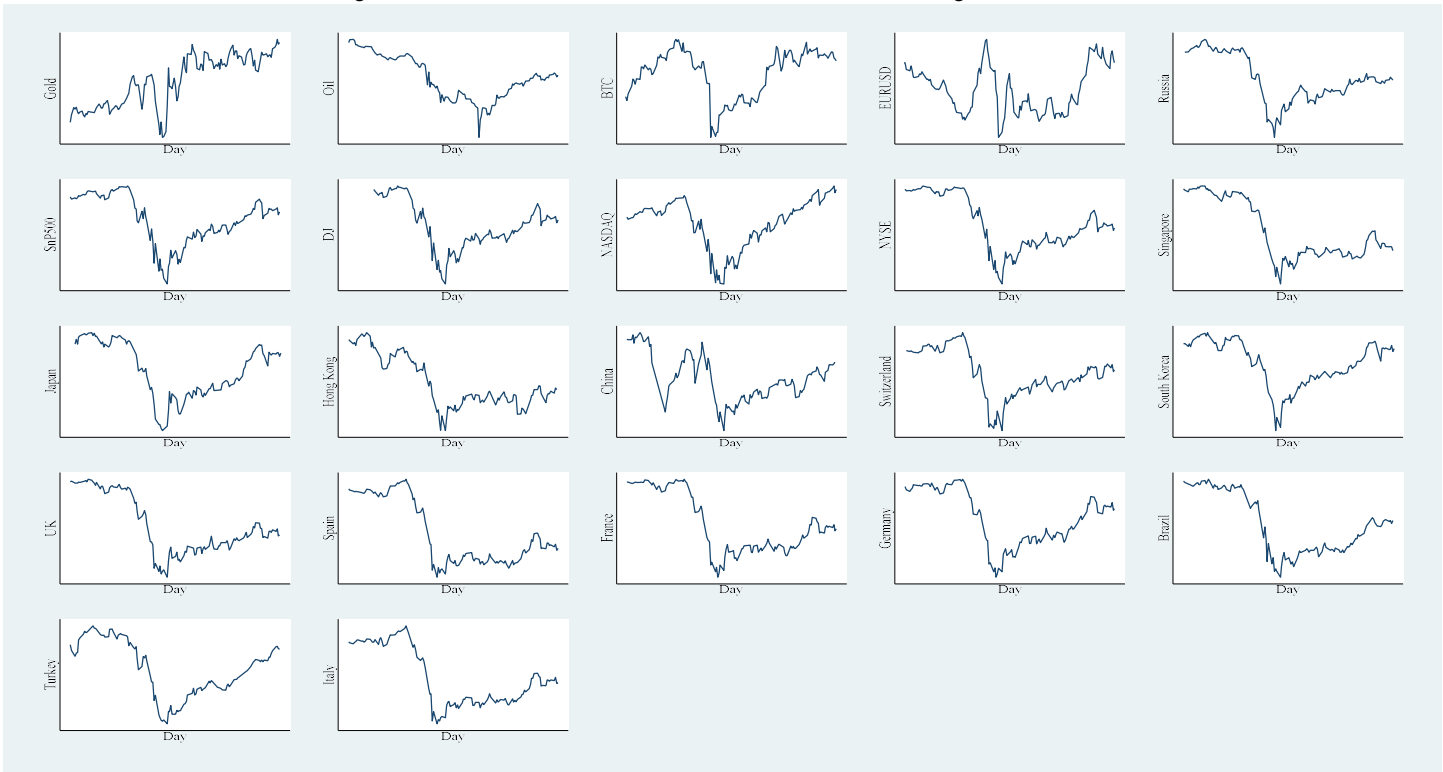

Supplement: Supplementary file 1 [file mmc1.pdf]
